# The Unusual Suspect: An Acute Gastric Dilation With Volvulus in a Scleroderma Patient

**DOI:** 10.7759/cureus.23436

**Published:** 2022-03-24

**Authors:** Anusha Agarwal, Alla Turshudzhyan, Alexander Khamechand, Michael Schuster, Micheal Tadros

**Affiliations:** 1 Internal Medicine, Albany Medical College, Albany, USA; 2 Internal Medicine, University of Connecticut Health, Farmington, USA; 3 Radiology, Albany Medical Center, Albany, USA; 4 Gastroenterology and Hepatology, Albany Medical Center, Albany, USA

**Keywords:** dysphagia, ssc, gastric volvulus, gastric dilation, scleroderma

## Abstract

Systemic scleroderma (SSc) is a chronic autoimmune disorder that can affect various organ systems. About 90% of patients with SSc have gastrointestinal (GI) manifestations, with esophageal dysmotility being the most frequently reported. While esophageal involvement is the most common, other segments of the upper GI tract can be affected as well, such as the stomach or small bowel. Some of the examples of gastric involvement include gastric antral vascular ectasia (GAVE) and gastroparesis. Small bowel involvement can present with reduced contractility, pseudo-obstruction, small intestinal bacterial overgrowth (SIBO), and atrophy. Although many of these manifestations bear little clinical urgency, acute gastric dilation or pseudo-obstruction constitute a medical emergency and require prompt intervention. We are presenting a case of acute gastric dilation with gastric volvulus in the setting of SSc, which is not well reported in the medical literature. We hope to increase providers’ awareness of this rare manifestation of SSc to facilitate prompt diagnosis and intervention. Furthermore, we hope to prompt more research to be done to better understand its pathophysiology and determine whether this manifestation of SSc is preventable.

## Introduction

Systemic scleroderma (SSc) is a chronic autoimmune disorder characterized by vascular and fibrotic changes affecting both the skin and internal organs. Approximately 90% of patients with SSc present with gastrointestinal (GI) manifestations, with esophageal dysmotility being the most frequently reported [1]. The most common symptoms on presentation are reflux, regurgitation, and dysphagia. This is likely due to decreased esophageal peristalsis and decreased lower esophageal sphincter tone. While the esophagus is the most commonly affected part of the GI tract in SSc patients, the stomach and small intestine can be affected as well. Gastric involvement may include the development of gastric antral vascular ectasia (GAVE) and decreased peristalsis, leading to gastroparesis. SSc may also precipitate small bowel hypomotility leading to pseudo-obstruction, small intestinal bacterial overgrowth (SIBO), and atrophy with resultant diverticula. Although some forms of upper GI tract involvement bear little clinical urgency, acute gastric dilation or pseudo-obstruction constitute medical emergencies and require prompt intervention. These rare but severe complications of long-standing SSc are associated with high mortality and are of clinical importance. We present a rare case of acute gastric dysmotility and dilation manifesting as acute pseudo-obstruction in the setting of long-standing SSc. This case was presented at the national American College of Gastroenterology conference in 2021.

## Case presentation

**Figure 1:**
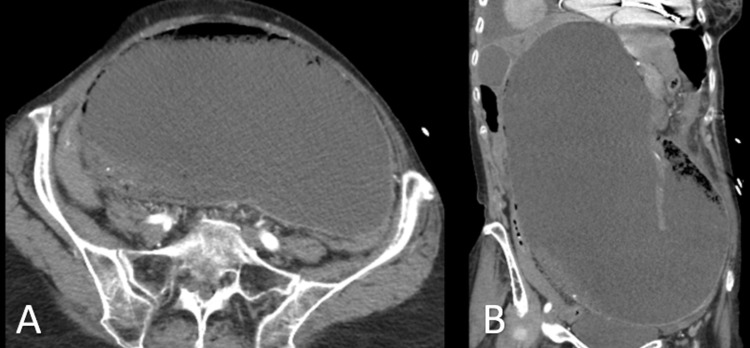
CT of the abdomen without contrast Imaging demonstrates markedly dilated stomach in (A) transverse view and in (B) coronal view.

A 72-year-old female with interstitial lung disease (ILD) and a well-controlled SSc on mycophenolate presented to the clinic with two days of worsening nausea, non-bloody emesis, and severe diffuse abdominal pain and distension. The patient had no prior abdominal surgeries. Prior workup had revealed a dilated esophagus, megaduodenum, and delayed gastric emptying, but symptoms were well controlled on proton pump inhibitors (PPIs) and a low residue diet. On presentation, laboratory results were remarkable for leukocytosis and lactic acidosis. An abdominal computed tomography (CT) revealed a markedly dilated stomach with a possible volvulus (Figure 1).

Laparotomy confirmed acute gastric dilation and volvulus, causing necrosis of the greater curvature of the stomach. Gastric volvulus correction with partial gastrectomy was performed with the subsequent placement of a PEG tube for decompression. The patient’s condition stabilized, and the PEG tube was later reversed.

## Discussion

Acute gastric dilation complicated by gastric volvulus in patients with SSc is not well documented in the medical literature. Its pathogenesis remains unclear. Based on our understanding of SSc pathophysiology, the likely underlying mechanism of acute gastric dilation in this patient is multifactorial and includes increased collagen deposition and fibrosis, auto-antibodies affecting myenteric neurons and neural cholinergic-mediated contraction, vascular ectasia, and decreased blood flow [2,3]. The characteristic SSc findings of collagen and elastic fibers surrounding smooth muscle cells under electron microscopy have been reported in the stomach [3]. Subsequently, the widened intercellular space contributes to gastroparesis by altering cellular contraction and the propagation of signals between the smooth muscle cells [3]. Additionally, recent studies suggest a role for autoantibodies against muscarinic 3 receptor (M3R) contributing to the pathogenesis of SSc [2,4]. Given the presence of M3R in the stomach, it can be postulated that the presence of autoantibodies against M3R may have contributed to the pathophysiology of gastric stasis in our patient [5,6]. The level of antibody titers against M3R correlated with increased GI manifestations in SSc patients, and immunoglobins directed at the autoantibodies were shown to minimize cholinergic dysfunction in patients [2].

It is also possible that acute gastric dysmotility and gastric volvulus in an SSc patient may follow a pathophysiologic mechanism similar to that of a small intestinal pseudo-obstruction. Some of the common precipitants of small intestinal pseudo-obstruction are electrolyte derangements, anticholinergic medications, and elevations in inflammatory cytokines. While our patient was not on any anticholinergic medications and did not have any electrolyte derangements, we wonder if inflammatory cytokines could have contributed to her presentation. Subsequent volvulus likely occurred around the attachment of the stomach to the gastrohepatic, gastrocolic, gastrosplenic, and gastrophrenic ligaments [7]. It is possible that the gastric dilation coupled with the patient’s severe disease presentation of SSc may have affected the laxity of ligaments anchoring the stomach in the peritoneum [8]. The resulting laxity of the ligaments likely gave rise to abnormal rotation of the stomach, which compromised the mesenteric vessels, leading to ischemia and necrosis along the greater curvature of the stomach [7,8].

Management options for gastric volvulus depend on the degree of gastric obstruction, presence of ischemia, and comorbid conditions of the patient. In the case of an acute presentation of gastric volvulus, initial management includes intravenous fluid resuscitation and nasogastric tube placement for decompression [9]. Definitive management follows and includes physically alleviating the obstruction, removing ischemic tissue, and anchoring the stomach to prevent further exacerbation [10]. Definitive management can be achieved via an endoscopic, laparoscopic, or open surgical approach [9]. Endoscopic or laparoscopic approaches may be better, less invasive alternatives to open surgical repair in patients with multiple comorbid conditions; however, there is a significant risk of gastric perforation [8]. In patients who present with a milder presentation of gastric volvulus and are clinically stable, immediate intervention may be postponed until patients are optimal surgical candidates based on their individual anatomy, preferences, and comorbid conditions [9].

## Conclusions

Acute gastric dilation and gastric volvuli are medical emergencies and manifestations of SSc that are not well reported in the available medical literature. We hope to increase providers’ awareness of this rare manifestation of SSc to facilitate prompt diagnosis and intervention. We also hope to prompt more research to be done to better understand its pathophysiology and determine whether this manifestation of SSc is preventable.
